# Validation of the Accuracy of Automatic Measurement of Blood Volume in Culture Bottles for Blood Culture

**DOI:** 10.3390/diagnostics13162685

**Published:** 2023-08-15

**Authors:** Kyoungbo Kim, Sunggyun Park

**Affiliations:** Departments of Laboratory Medicine, Keimyung University School of Medicine, Daegu 42601, Republic of Korea; kimbo707@dsmc.or.kr

**Keywords:** bloodstream infection, blood culture, blood volume

## Abstract

Several manufacturers have developed systems that automatically measure the amount of blood in culture bottles. We compared the volumes measured automatically by the Virtuo instrument (bioMérieux, France) (height-based volumes) and those calculated by weighing the bottles. In all, 150 pairs of aerobic and anaerobic blood culture bottles (BacT/ALERT FA/FN Plus, bioMérieux) were randomly selected over two periods to compare the height- and weight-based volumes and analyze the effect of foam. We also estimated the limit of detection (LOD) and the cut-off value for 5 mL equine blood. The mean height-based volume was approximately 0.67 mL greater than the weight-based volume, particularly in anaerobic culture bottles. Foam did not have a significant effect. The LOD for the automatic height-based volume of equine blood was 0.2–0.4 mL. The 5 mL cut-off was 4–4.2 mL. Therefore, when reporting or monitoring blood volume within culture bottles in the laboratory, these performance characteristics should be adequately considered.

## 1. Introduction

Bloodstream infection (BSI) is a life-threatening medical emergency with a mortality rate of up to 30%, even when treated with empirical antibiotics [1]. In South Korea, the 30-day mortality rates of BSIs caused by Escherichia coli and Klebsiella pneumoniae are 10% and 16.9%, respectively, as in other countries [2]. The gold-standard diagnostic test for BSI remains blood culture, but the diagnostic yield depends on clinical factors and the blood collection and transportation methods [3]. An adequate blood volume is very important; the pathogen loads in most BSI patients are very low [4,5]. The Clinical and Laboratory Standards Institute (CLSI) strongly recommends visually inspecting or weighing blood culture bottles [6]. Several major manufacturers have developed systems that automatically measure the amounts of blood in culture bottles. The BACTEC FX instrument (Becton-Dickinson Microbiology Systems, Sparks, MD, USA) measures blood volume by measuring the CO_2_ production of red blood cells [7,8]. There are several comparative evaluations of the blood volume measurement for the BACTEC FX instrument, and they report a difference of approximately 0.2–0.3 mL compared to the weight-based volume [8,9]. The BacT/Alert Virtuo instrument (bioMérieux, Marcy l’Étoile, France) measures blood volume by reference to the liquid height. The manufacturer of the Virtuo instrument has not provided concrete reliability evidence regarding height-based blood volume measurements. To date, there has been only one comparative study reported on the blood volume measurement of the BacT/Alert Virtuo instrument [10]. According to Lee et al., a relatively substantial difference was reported compared to the previous study using BACTEC FX instrument comparing it with the weight-based volume. That difference was more pronounced in the aerobic bottle. Indeed, upgrades to the BacT/Alert Virtuo instrument and software are being consistently conducted. Considering that the previous study used Virtuo instrument older version, a comparative evaluation using the new version of the instrument is necessary. So, we compared this height-based volume using the new version of the BacT/Alert Virtuo instrument to the weight-based volume. Additionally, we aimed to provide the blood volume along with culture results to assist in the interpretation of blood culture results. Therefore, we used equine blood to conduct a more accurate performance validation of the BacT/Alert Virtuo instrument including limit of blank, limit of detection, and precision estimation.

## 2. Materials and Methods

### 2.1. Sample Collection

**Figure 1:**
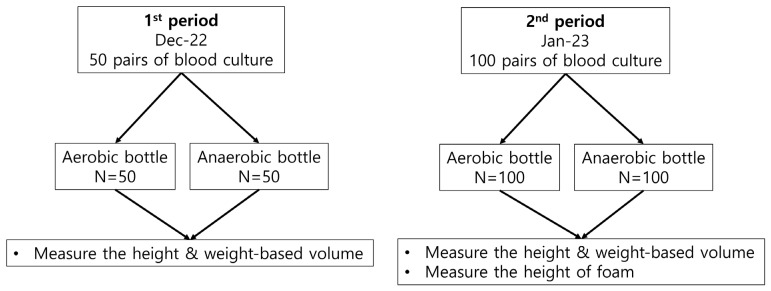
Work performed during two different periods on automatic measurements of blood volumes in culture bottles.

In total, 150 pairs of aerobic and anaerobic blood culture bottles (BacT/ALERT FA/FN Plus, bioMérieux) were randomly selected over two periods (Figure 1). During the first period (1 week in December 2022), 50 pairs of bottles were randomly selected to compare the height- and weight-based volumes. During the second period (1 week in January 2023), 100 pairs of culture bottles were randomly selected to analyze the effect of foam on Virtuo performance. The study was approved by the Institutional Review Board of Dongsan Medical Center (approval no. DSMC 2023–02–049).

### 2.2. Measurement of Blood Volumes and Foam

To measure height-based volumes, we used the Virtuo instrument version 3.0.01.1274 running Myla middleware (version 4.7.1). After each bottle was loaded on the instrument, the height-based volume was shown on the screen. The weight-based volume was calculated by subtracting the average weight of 20 empty aerobic and anaerobic bottles from the weight of bottles filled with blood from the same lot. In line with Lee et al., the weight-based volume was calculated by dividing the value obtained by subtracting the weight of empty bottles from that of bottles with blood by 1.055, to reflect the specific gravity of blood [10]. During the second period, the presence/absence of foam was checked by the naked eye just before height-based volume estimation.

### 2.3. Limit of Blank, Limit of Detection, and Cut-Off of the Height-Based Volume

We have validated the limit of blank (LOB) for both the weight-based and height-based volume measurement method. For the weight-based volume measurement, we measured the weight of 20 empty aerobic bottles and 20 empty anaerobic bottles with the same lot number. As for the height-based volume measurement, we performed three repetitions of volume measurements using Virtuo instrument on 20 empty aerobic bottles and 20 empty anaerobic bottles. The LOB was determined following the classical non-parametric method specified in CLSI EP17-A2 (alpha = 0.05) [11].

We used defibrinated equine blood (Synergy Innovation, Seongnam, Republic of Korea) to estimate the limit of detection (LOD) of height-based volume. We prepared three aerobic and three anaerobic culture bottles containing equine blood volumes of 0.2 to 1.2 mL at 0.2 mL intervals. The LOD was the lowest volume yielding no more than one 0 mL readout when six measurements for each volume were taken.

We also estimated the 5 mL height-based volume cut-off by measuring seven volumes of equine blood from 4 to 5.2 mL at 0.2 mL intervals in two aerobic and two anaerobic bottles. The cut-off was the lowest volume yielding no more than two readouts of 5 mL or more when four measurements were performed.

We also aimed to assess the precision of the volume measurement by utilizing data obtained from repeated measurements of the equine blood sample at the same volume for estimation of LOD and 5 mL height-based volume cut-off. Based on this, we intended to determine the reporting of blood volume in conjunction with the blood culture results in our laboratory.

### 2.4. Statistical Analyses

Since the height-based volumes are provided rounded to zero decimal places, we used all the weight-based volumes in the same manner, rounding them to zero decimal places as well. The height- and weight-based volumes were subjected to Deming and Passing Bablok regressions. The Wilcoxon signed-rank test was used to confirm the mean differences between weight-based and height-based volumes in aerobic, anaerobic, and both bottles. For qualitative evaluation, height- and weight-based volumes were divided into two groups (high- and low-volume) using both 5 mL and 8 mL cut-offs and the extents of agreement and the Cohen kappa coefficients were determined [12]. The differences in the height- and weight-based volumes of samples with and without foam were compared using the Mann–Whitney U-test.

In order to combine the coefficient of variation from various levels of inoculated volume, we performed the decomposition of means and standard deviations. To accomplish this we utilized the online tool available at https://www.statstodo.com/CombineMeansSDs.php (accessed on 8 June 2023). We have set a criterion for reporting the blood volume in conjunction with the blood culture results, which states that the coefficient of variation should be below 20%.

## 3. Results

### 3.1. Height- vs. Weight-Based Volumes

**Figure 2:**
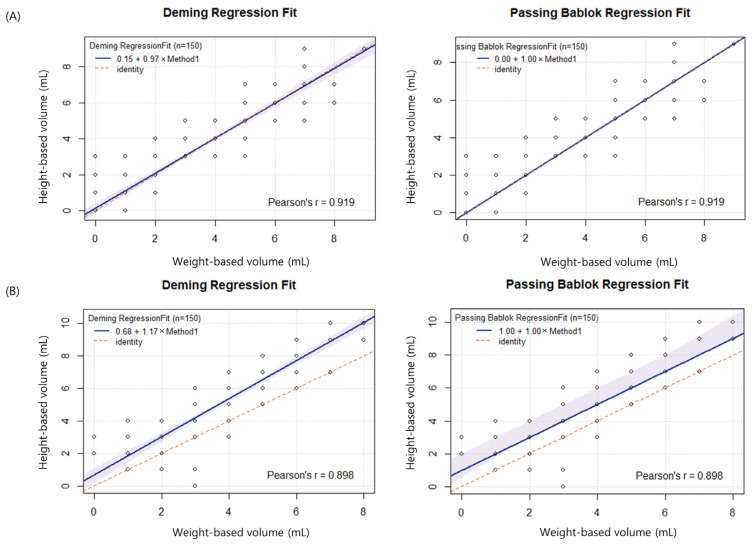
Correlations between weight- and height-based volumes in aerobic (**A**) and anaerobic (**B**) bottles.

The mean height-based volume (300 bottles) was approximately 0.67 mL greater than the weight-based volume (Table 1). The difference was higher for anaerobic bottles and attained statistical significance. No significant differences were observed between the height- and weight-based volumes in aerobic bottles. The Pearson correlation coefficient for aerobic bottles was higher than that for anaerobic bottles (Figure 2).

In qualitative evaluations of the high- and low-volume groups using 5 mL cut-off, the overall agreement was approximately 87% (Table 2). Again, the agreement was better for aerobic than anaerobic bottles.

In qualitative evaluations of the high- and low-volume groups using 8 mL cut-off, the overall agreement was approximately 94% (Table 3). The agreement exceeded 90% for both aerobic and anaerobic bottles. However, we found only five bottles (3 aerobic and 2 anaerobic bottles) with volume exceeding 8 mL in weight-based volume and the Cohen kappa coefficients were lower than that using the 5 mL cut-off.

### 3.2. Effect of Foam

**Figure 3:**
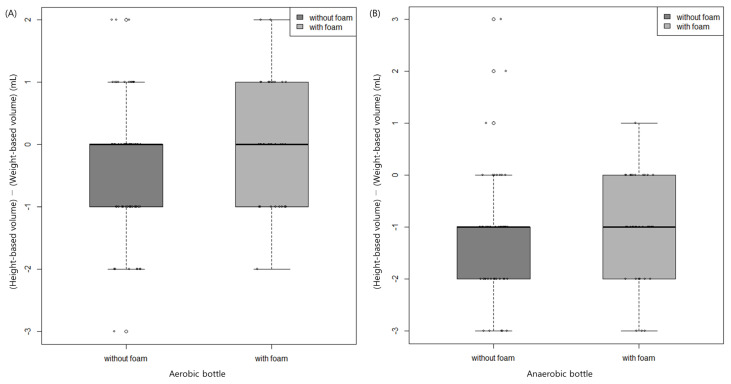
The difference between height-based volume and weight-based volume by foam status (yes/no) in aerobic (**A**) and anaerobic (**B**) bottles. Data are medians with interquartile range.

Of the 200 culture bottles (100 aerobic and 100 anaerobic) evaluated in the second period, 33 aerobic and 40 anaerobic bottles evidenced foam of median height 0.4 cm (range 0.2–0.6 cm) in both bottles (Supplementary Tables S1 and S2). The weight- and height-based volumes did not significantly differ by foam status (yes/no) (Figure 3).

### 3.3. Limit of Blank, Limit of Detection, and Cut-Off Value of Height-Based Volume

The weight-based volume for all 20 empty aerobic and anaerobic bottles resulted in 0 mL when rounded to zero decimal places (Table 4, Supplementary Table S3). Therefore, the LOB for the weight-based volume can be estimated as 0 mL. Similarly, for the height-based volume using aerobic bottles, the LOB was also found to be 0 mL when using the data from the three repetitions of measurements on each of the 20 empty aerobic bottles. However, when using the empty anaerobic bottles for the height-based volume measurement, the LOB was determined to be 2 mL.

The LOD for the height-based volume of equine blood was 0.2–0.4 mL (Supplementary Table S4). The 5 mL cut-off for the height-based volume was 4–4.2 mL (Supplementary Table S5). There were no significant differences between the added and height-based volumes in either aerobic or anaerobic bottles (Supplementary Figure S1).

When evaluating the precision based on repeated measurements of the equine blood at the same volume, it was observed that the coefficient of variation was approximately 75% for inoculated equine blood volume ranging from 0.2 mL to 1.2 mL (Supplementary Table S4). This value exceeds the reporting criterion of 20% set for the coefficient of variation. On the other hand, for inoculated equine blood volumes ranging from 4 mL to 5.2 mL, the coefficient of variation was approximately 19.6% (Supplementary Table S5). This indicates a lower coefficient of variation compared to the lower volume range.

## 4. Discussion

Although automatic blood culture systems are widely used, culture may fail if pathogen numbers are low in BSI patients [4,5]. The blood volume is thus a very important parameter. Although the CLSI recommends culturing 20–30 mL blood, the mean volume was only 7.7 mL in nine university hospitals of Korea [6,13]. The CLSI recommends diluting blood in broth with a blood-to-broth ratio of 1:5 to 1:10 to reduce the concentrations of various substances that inhibit the growth of blood microorganisms [6]. Therefore, most manufacturers of blood culture bottles recommend adding 8–10 mL blood [14]. However, many studies have found that the average volume is in fact less than 5 mL [13,14]. In our study, we also found that the bottles containing blood equal to or exceeding 8 mL accounted for only 1.67% of the total blood culture bottles. Therefore, it is challenging to determine the appropriateness of the blood volume with a cut-off of 8–10 mL in an actual clinical setting. In this study, we used the average volume of 5 mL, which represents the actual blood volume per culture bottle in clinical setting, as a reliable minimum threshold for the blood volume. Thus, we adopted a 5 mL cut-off when dividing samples into those of high and low volume.

Several blood culture systems automatically measure blood volumes, including our BacT/Alert Virtuo instrument. However, the manufacturer of the Virtuo instrument has not provided concrete reliability evidence regarding height-based blood volume measurements. Therefore, we compared these height-based volumes to weight-based volumes. In an earlier study, the differences were 1.4 mL for aerobic and 0.21 mL for anaerobic bottles [10]. In this study, the figures were 0.05 and 1.29 mL. Thus, we found a much greater difference for anaerobic than aerobic bottles. This may be attributable to the Virtuo software employed. In the previous study, version 02.01.06.928 was used; we employed the upgraded version 03.01.01.1274. The version 3.0 update incorporated various improvements, including enhancement of the accuracy of blood volume measurements. Despite such improvements, we found that height-based volumes in anaerobic bottles were significantly greater than those in aerobic bottles. We explored whether this might be attributable to foam; this was not the case. As the sample size was small and the amounts of foam were low, further research should explore the effect of foam on the measurement of height-based volumes.

Foam aside, the bottles contain broth in which adsorbent polymeric beads are suspended. If the suspension partially dries, leaving a rim of beads around the original meniscus, it is possible that the instrument records that rim as the sample height. The larger volume of broth in anaerobic bottles compared to aerobic bottles, and the higher LOB observed in height-based volume measurements using anaerobic bottles in our study, corroborate that the broth in the bottle can affect the accuracy of the height-based volume measurement. However, considering the principle of height measurement using the Virtuo instrument based on light sources, it is essential to acknowledge that height-based volume measurement using empty bottles may be less accurate compared to using bottles filled with blood.

Although the sample size was small, the LOD for height-based volume measurements of equine blood was 0.2–0.4 mL and the 5 mL cut-off was 4–4.2 mL. Thus, if 0.2 mL blood is added, this will very likely be reported as 1 mL, and 4 mL blood may be read as 5 mL. In contrast to our results for human blood samples, no significant difference between the aerobic and anaerobic bottles was observed when employing equine blood, perhaps attributable to differences in blood collection and bottle-filling methods. Further research is required. However, the equine blood experiments revealed limitations in the clinical applicability of height-based volume measurements, including failure to detect concentrations above the LOD given the poor precision, and significant differences in the height-based volumes in bottles with the same amounts of blood.

Indeed, ensuring an adequate level of precision is crucial for reporting the results of any test. The accuracy and reliability of the reported data greatly depend on the precision of the measurements performed. There is no standardized value for the coefficient of variation that is considered suitable for reporting the blood volume in blood culture bottles. It varies depending on the test method, purpose of the test, and clinical context. Generally, coefficient of variation values below 5% are often associated with good performance, whereas values above 10% are associated with poor performance [15]. We have established a criterion of 20% or lower, taking into account both the number of repeated measurements and the clinical significance of the test results. Although we were unable to perform an adequate number of repeated measurements, the coefficient of variations for the blood volume measurements using the BacT/Alert Virtuo instrument were approximately 75% for the range of 0.2–1.2 mL and 19.6% for the range of 4–5.2 mL. These coefficients of variation indicate that that there is an imprecision of more than 10% in the measurements. The observed high imprecision can cause errors in measuring the blood volume in culture bottles where the amount of blood exceeds the limit of detection but cannot be quantified. It can also lead to misinterpretation when a volume below 5 mL of blood is inoculated, falsely assuming that sufficient volume of blood has been added. However, considering that the coefficient of variation for the meaningful blood volume cut-off of 5 mL was relatively low and below 20% in this study, it may be worth considering reporting the blood volume. Understanding the limitations of the test’s precision can be helpful in interpreting the results of blood culture and blood volume.

Our work had several limitations. First, we had fewer specimens than previous studies did. Second, we did not explore factors that might affect the height-based volume other than foam, such as the sample collection and transportation methods, transportation time, and the time from sample collection to instrument loading. Third, given the low numbers of samples used to establish the LOD and 5 mL cut-off, only approximate values are presented. Fourth, the manufacturer of the Virtuo instrument does not provide appropriate control material for blood volume measurements. Therefore, the tests conducted in this study were carried out without internal quality control. Through experiments using equine blood, the high imprecision of blood volume measurements with the Virtuo instrument was confirmed. Thus, it became evident that performing quality control using suitable control material like equine blood is essential. Fifth, due to ethical concerns, we were unable to conduct experiments by administering a specific volume of human blood arbitrarily and measuring the volume using the Virtuo instrument. Instead, we used commercial equine blood for verification. However, we could not validate the correlation between the performance of using equine blood and human blood. Nevertheless, considering that the Virtuo instrument measures the volume of injected blood based on height, if the same volume were administered, we believe that the measurement values for human and equine blood would not be significantly different.

Despite these limitations, this study was the first to explore automatic blood volume measurements using the Virtuo instrument (version 3.0) and to investigate whether foam affects height-based volume. The Virtuo instrument can be used to measure blood volumes for aerobic blood culture. However, it has been confirmed that the measurement of blood volume in anaerobic blood culture bottles is imprecise and significantly overestimates the actual blood volume. Therefore, it is evident that there are limitations to including the blood volume in the reporting of blood culture results.

## 5. Conclusions

The blood volume measured using the Virtuo instrument is approximately 0.67 mL higher than the volume measured based on weight, and this difference is more pronounced in anaerobic bottles. Additionally, experimental results using equine blood showed an imprecision of over 10%. Therefore, when reporting or monitoring blood volume within culture bottles in the laboratory, these performance characteristics should be adequately considered.

## Figures and Tables

**Table 1:** Height- and weight-based volumes of 300 bottles.

Type (No.)	Weight-Based Volumes (Mean, mL)	Height-Based Volumes (Mean, mL)	Difference (mL)	*p*-Value
Aerobic (150)	3.71	3.76	0.05	0.5693
Anaerobic (150)	3.61	4.9	1.29	<2.2 × 10^−16^
Total (300)	3.66	4.33	0.67	<2.2 × 10^−16^

**Table 2:** Extents of agreement between the high- and low-volume groups using 5 mL cut-off.

Type (No.)	No. of High Weight-Based Volumes (≥5 mL)		No. of Low Weight-Based Volumes (<5 mL)		Agreement (95% CI)	Kappa
	No. of High Height-Based Volumes (≥5 mL)	No. of Low Height-Based Volumes (<5 mL)	No. of High Height-Based Volumes (≥5 mL)	No. of Low Height-Based Volumes (<5 mL)		
Aerobic (150)	56	11	5	78	0.893 (0.834–0.933)	0.782
Anaerobic (150)	61	0	24	65	0.84 (0.773–0.890)	0.688
Total (300)	117	11	29	143	0.867 (0.824–0.901)	0.732

Abbreviations: CI, confidence interval.

**Table 3:** Extents of agreement between the high- and low-volume groups using 8 mL cut-off.

Type (No.)	No. of High Weight-Based Volumes (≥8 mL)		No. of Low Weight-Based Volumes (<8 mL)		Agreement (95% CI)	Kappa
	No. of High Height-Based Volumes (≥8 mL)	No. of Low Height-Based Volumes (<8 mL)	No. of High Height-Based Volumes (≥8 mL)	No. of Low Height-Based Volumes (<8 mL)		
Aerobic (150)	1	2	5	142	0.953 (0.907–0.977)	0.201
Anaerobic (150)	2	0	11	137	0.927 (0.873–0.959)	0.249
Total (300)	3	2	16	279	0.94 (0.907–0.962)	0.23

Abbreviations: CI, confidence interval.

**Table 4:** Limit of blank of weight-based volume measurement and height-based volume measurement.

Type (No.)	LOB of Weight-Based Volumes (mL)	LOB of Height-Based Volumes (mL)
Aerobic (20)	0	0
Anaerobic (20)	0	2

Abbreviations: LOB, limit of blank.

## Data Availability

The data presented in this study are available on request from the corresponding author.

## Supplementary Materials

The following supporting information can be downloaded at: https://www.mdpi.com/article/10.3390/diagnostics13162685/s1, Table S1: Weight-based volume, height-based volume and height of foam in aerobic bottles; Table S2: Weight-based volume, height-based volume and height of foam in anaerobic bottles; Table S3: Results for limit of blank using empty blood culture bottles; Table S4: Results for estimating limit of detection using equine blood; Table S5: Results for estimating cut-off value of 5 mL using equine blood; Figure S1: Difference between height-based volume and inoculated volume in aerobic and anaerobic bottles.
