# Supplementary material for: Validation of the Accuracy of Automatic Measurement of Blood Volume in Culture Bottles for Blood Culture
Source: Diagnostics (Basel). 2023 Aug 15;13(16):2685. doi: 10.3390/diagnostics13162685 (PMC10453367; doi:10.3390/diagnostics13162685)
Supplement: Supplementary file 1 [file diagnostics-13-02685-s001.zip › Supplementary Fiqure.pptx]

## Slide 1
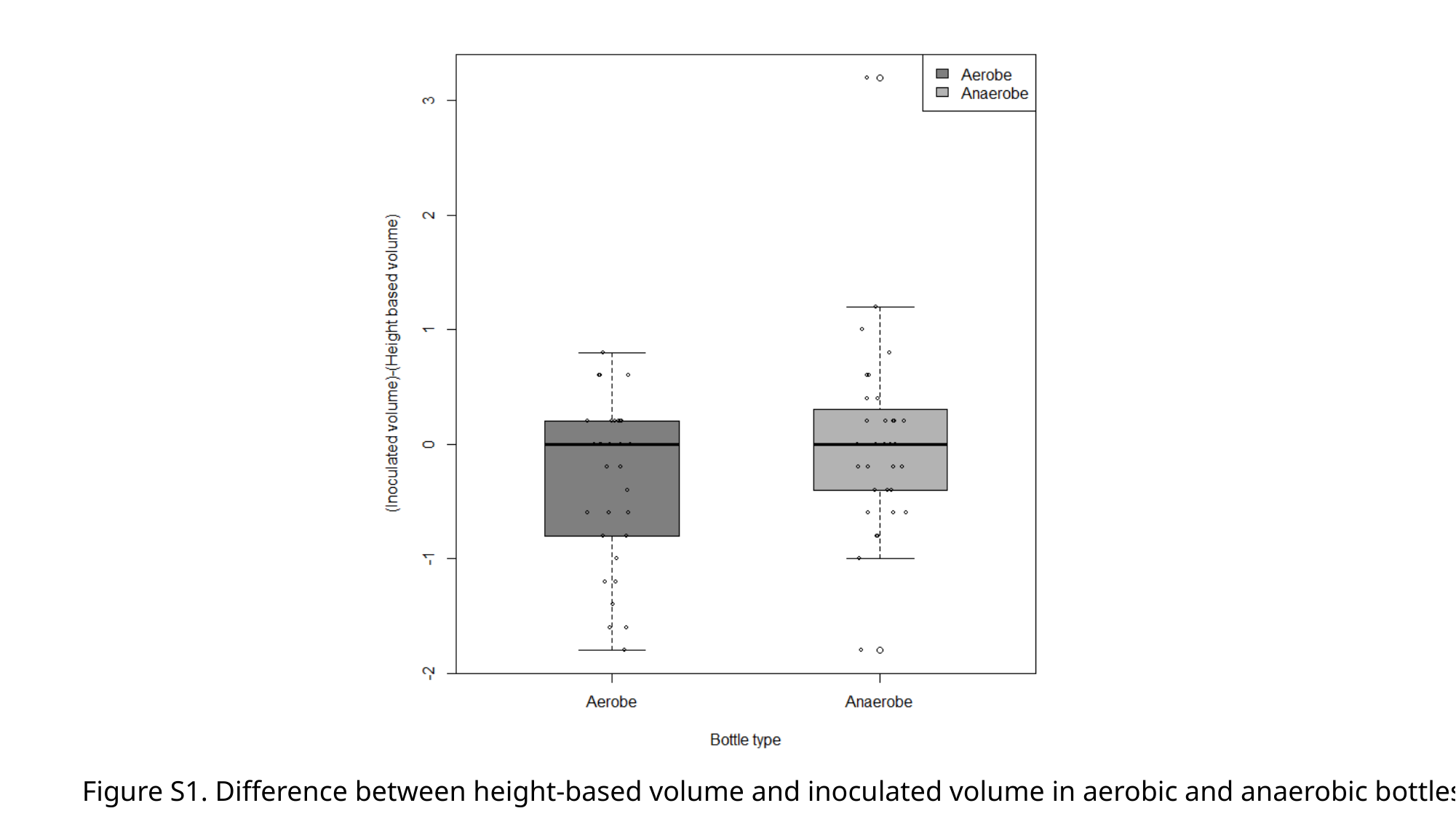

Figure S1. Difference between height-based volume and inoculated volume in aerobic and anaerobic bottles
